# Cytoprotective Effect of Idebenone through Modulation of the Intrinsic Mitochondrial Pathway of Apoptosis in Human Retinal Pigment Epithelial Cells Exposed to Oxidative Stress Induced by Hydrogen Peroxide

**DOI:** 10.3390/biomedicines10020503

**Published:** 2022-02-21

**Authors:** Maria Elisabetta Clementi, Michela Pizzoferrato, Giada Bianchetti, Anna Brancato, Beatrice Sampaolese, Giuseppe Maulucci, Giuseppe Tringali

**Affiliations:** 1 Institute of Chemical Sciences and Technologies “Giulio Natta” (SCITEC)-CNR, Largo F. Vito 1, 00168 Rome, Italy; elisabetta.clementi@scitec.cnr.it (M.E.C.); beatrice.sampaolese@scitec.cnr.it (B.S.); 2 Pharmacology Section, Department of Health Care Surveillance and Bioethics, Università Cattolica del Sacro Cuore, Largo F. Vito 1, 00168 Rome, Italy; michela.pizzoferrato@gmail.com; 3 Fondazione Policlinico Universitario Agostino Gemelli IRCSS, 00168 Rome, Italy; giada.bianchetti@unicatt.it (G.B.); giuseppe.maulucci@unicatt.it (G.M.); 4 Biophysics Section, Neuroscience Department, Università Cattolica Del Sacro Cuore, Largo F. Vito 1, 00168 Rome, Italy; 5 Department of Health Promotion, Mother and Child Care, Internal Medicine and Medical Specialties of Excellence “G. D’Alessandro”, University of Palermo, 90127 Palermo, Italy; anna.brancato@unipa.it

**Keywords:** idebenone, ARPE-19 (human-RPE cell line), mitochondria, oxidative stress, nuclear factor erythroid 2-related factor (Nrf2), apoptosis

## Abstract

Idebenone is a ubiquinone short-chain synthetic analog with antioxidant properties, which is believed to restore mitochondrial ATP synthesis. As such, idebenone is investigated in numerous clinical trials for diseases of mitochondrial aetiology and it is authorized as a drug for the treatment of Leber’s hereditary optic neuropathy. Mitochondria of retinal pigment epithelium (RPE) are particularly vulnerable to oxidative damage associated with cellular senescence. Therefore, the aim of this study was to explore idebenone’s cytoprotective effect and its underlying mechanism. We used a human-RPE cell line (ARPE-19) exposed to idebenone pre-treatment for 24 h followed by conditions inducing H_2_O_2_ oxidative damage for a further 24 h. We found that idebenone: (a) ameliorated H_2_O_2_-lowered cell viability in the RPE culture; (b) activated Nrf2 signaling pathway by promoting Nrf2 nuclear translocation; (c) increased Bcl-2 protein levels, leaving unmodified those of Bax, thereby reducing the Bax/Bcl-2 ratio; (d) maintained the mitochondrial membrane potential (ΔΨ_*m*_) at physiological levels, preserving the functionality of mitochondrial respiratory complexes and counteracting the excessive production of ROS; and (e) reduced mitochondrial cytochrome C-mediated caspase-3 activity. Taken together, our findings show that idebenone protects RPE from oxidative damage by modulating the intrinsic mitochondrial pathway of apoptosis, suggesting its possible role in retinal epitheliopathies associated with mitochondrial dysfunction.

## 1. Introduction

Idebenone (IDB) is a cognitive nootropic and antioxidant agent. Its free radical scavenger properties and its ability to modulate and restore the production of ATP at the mitochondrial level, as well as its capacity to protect membranes against lipid peroxidation, are well known [1]. However, recent studies, conducted on different experimental models, have broadened the range of action mechanisms underlying the pleiotropic cytoprotective effects of IDB. It has been shown that IDB: up-regulates the RNA binding protein Lin-28 homolog A; inhibits the functions of the p52Shc protein; activates the Akt kinase; acts as a selective agonist of PPARα/γ receptors; prevents LNRP3 inflammasome activation under hypoxia-reperfusion conditions; and down-regulates endoplasmic reticulum stress markers [2]. Due to these characteristics, IDB has been studied and used for many years in various fields of medicine and in pharmaceuticals, cosmetics, and as a supplement in food. 

IDB’s effects have been investigated in both pre-clinical and clinical trials for the treatment of Alzheimer’s disease [3,4,5] and cognitive-behavioral deficits of vascular and degenerative origin [6,7], as well as for therapies for some rare hereditary pathologies characterized by mitochondrial damage, such as Duchenne muscular dystrophy and Friedreich ataxia, with mixed success [8,9,10]. The marketing authorization of Raxone^®^ (a drug containing the active ingredient IDB) for the treatment of Leber’s hereditary optic neuropathy (LHON) [11], a neurodegenerative disease of the optic nerve characterized by the sudden loss of vision in adults and adolescents, rekindled the spotlight on IDB and its possible therapeutic role in various ophthalmic diseases, especially those with a close connection between low vision and mitochondrial dysfunction [12,13].

The retinal pigment epithelium (RPE) is a monolayer of epithelial cells containing a dark-colored pigment (fuscin) that absorbs light, preventing it from spreading. Its position, between the photoreceptors and the cariocapillary vessels, is of fundamental importance. It acts as a dynamic barrier, serving the dual function of providing metabolic and functional support to the outer segment of photoreceptors, as well as allowing the passage of nutritional elements and the release of neurotrophic and growth factors from the choroid to the retina and vice versa [14,15,16]. RPE cells exhibit a greater adaptive response and resistance to oxidative stress than other cell types, although the mitochondria appear to be the limiting factor of this resistance [17]. In fact, mitochondria in RPE cells are particularly vulnerable to the oxidative damage associated with aging and exposure to endogenous and exogenous stressors [18,19]. In particular, mitochondrial DNA (mtDNA) is a critical cellular target for ROS, and it is known that oxidative damage to mtDNA is normally more persistent and severe than damage to nuclear DNA [20]. Oxidative stress-induced mtDNA damage is linked to mitochondrial dysregulation and cell death, characterized by reduced ATP production, increased ROS release, impaired calcium ion flux, a reduction in mitochondrial membrane potential, and leakage of pro-apoptotic factors into the cytoplasm, such as cytochrome c [21,22]. In light of the above and literary evidence on the subject [23,24], which supports a correlation between mitochondrial dysfunction and the aetiology of some retinal diseases, we were interested to investigate whether there is a link between the cytoprotective effect and the ability of IDB to support the homeostasis of mitochondria in situations of oxidative stress at the RPE level. 

Arend and Coll [25] have shown that IDB protects RPE cells from apoptosis by stabilizing the Bax/Bcl-2 ratio, in conditions of acute oxidative stress (2 h) induced by H_2_O_2_. With this in mind, the present study aims to obtain a more comprehensive understanding of the mechanisms regulating the IDB cytoprotective effect in the RPE at both the cytoplasmic and mitochondrial levels. We used an in vitro experimental model, previously developed by our laboratory, in which RPE cells (human ARPE-19 cell line) were exposed to H_2_O_2_ for 24 h [26]. The correlation between the RPE cells pre-treated with IDB (24 h) and cell viability, direct stimulation of Nrf2, as well as the modulation of the Bcl-2 family proteins (Bax and Bcl-2), were examined in our experimental paradigm. Furthermore, we investigated the possible mitochondrial support associated with the administration of IDB by analyzing its effects on the mitochondrial membrane potential, on the respiratory chain complexes, and on the production of mitochondrial ROS. Finally, in the same experimental context, we also investigated the effects of IDB on the release of cytochrome c and on the activity of caspase-3 in conditions of oxidative stress. 

Our results show that the cytoprotective effect of IDB on RPE exposed to oxidative stress involves the intrinsic mitochondrial pathway of apoptosis.

## 2. Materials and Methods

### 2.1. Cell Culture and Treatments

ARPE-19 cells were purchased from the American Type Cell Culture (ATCC-CRL-2302, Manassas, VA, USA). The cells were cultured in a DMEM/F12 medium (Sigma-Aldrich, St. Louis, MI, USA) supplemented with 10% FBS (Gibco; Thermo Fisher Scientific Inc., Waltham, MA, USA), L-Glutamine 2mM (Sigma-Aldrich, St. Louis, MI, USA), and 100 U/mL penicillin–streptomycin (Thermo Fisher Scientific Inc., Waltham, MA, USA) at 37 °C in a 5% CO_2_ environment. Once 80% confluence was achieved, the cells were subcultured at a concentration of 30,000 cells/cm^2^. 

On the day of the experiment, the cells were plated in multiwell plates or Petri dishes, when appropriate. All the experiments were performed with a serum-free medium. The experimental paradigm involved a 24 h pre-incubation period, during which the cells were treated with Idebenone (IDB) (treated groups) or medium alone (control group). This was followed by a second incubation period of 24 h, during which either H_2_O_2_ (Sigma-Aldrich, St. Louis, MI, USA) alone (to assess the oxidative damage) or H_2_O_2_ in the presence of readded IDB (to assess IDB’s effects in the presence of oxidative stress) was added to the incubation medium. For the IDB treatment medium, IDB powder (Sigma-Aldrich, St. Louis, MI, USA) was dissolved in DMSO to obtain a 10 mM stock solution. Further dilutions were made in the incubation medium, as necessary. Fresh solutions were prepared for each experiment. 

### 2.2. Experimental Conditions and Cell Availability

Dose response-curve experiments were conducted to determine the H_2_O_2_ and IDB concentrations for use in the study. For this purpose, ARPE-19 cells were plated in 96-well plates at a density of 15,000 cells/well and incubated for 24 h, with different concentrations of H_2_O_2_ (range 200–800 µM) and IDB (range 0.01–10 µM), the latter in the presence or absence of H_2_O_2_ (300 µM). Cell survival was evaluated by MTS assay (Promega, Waltham, MA, USA), according to the manufacturer’s instructions, by measuring absorbance at 490 nm using a microplate photometer (Victor 4, PerkinElmer, Waltham, MA, USA). Results are expressed as the percentage of cell viability relative to the untreated control. Furthermore, the morphological features of the cells were observed and photographed under all experimental conditions using a phase-contrast microscope (TE300-Eclipse-microscope; Nikon Corporation, Tokyo, Japan) at a magnification of ×20.

The experimental conditions used in this study were determined based on the viability of the results obtained: pre-treatment with 1 µM IDB for 24 h and subsequent treatment with H_2_O_2_ 300 µM for a further 24 h.

### 2.3. Quantification of Nuclear Activation of Nrf2

Nuclear activation of Nrf2 was determined by reference to nuclei purified from ARPE-19 cells treated as per the experimental protocol, using the nuclear extraction kit (ab113474-Abcam; Abcam Biomedical Campus, Discovery Drive, Trumpington, Cambridge CB2 0AX, UK) [27]. After measuring the protein concentration (Bio-Rad Assay Protein, Hercules, CA, USA), 20 µg of nuclear protein was added to the 96-well plate provided with the Nrf2 transcription factor assay kit (ab207223-Abcam; Abcam Biomedical Campus, Discovery Drive, Trumpington, Cambridge CB2 0AX, UK), according to the manufacturer’s instructions. The assay is a sandwich ELISA. An HRP-conjugated secondary antibody provides a sensitive colorimetric reading at 450 nm OD. Results are expressed as a percentage compared with the untreated control.

### 2.4. Bax and Bcl-2 Detection

To determine the apoptotic potential of ARPE-19, we assayed protein Bax and Bcl-2 with cell-based colorimetric ELISA kits (Assay Biotechnology, Sunnyvale, CA, USA) [28]. Briefly, quenching buffer, blocking buffer, and primary antibodies (rabbit polyclonal anti-Bax, rabbit polyclonal anti-Bcl-2, and mouse monoclonal anti-GAPDH) were added sequentially to the cells, seeded (at a density of 1.5 × 10^4^ cells/well) in a 96-well plate, and treated according to the experimental design. After incubation at 4 °C overnight, peroxidase-conjugated secondary antibodies (HRP-conjugated anti-rabbit IgG for Bax and Bcl-2; HRP-conjugated anti-mouse IgG for GADPH) were added. At the end of the incubation period, the plate was read with a microplate reader (BioTek Instruments, Winooski, VT, USA) at 450 nm. All values obtained, normalized to GAPDH OD 450, are expressed as a percentage compared with the untreated control.

### 2.5. Confocal Microscopy Imaging and Evaluation of Mitochondrial Membrane Potential

Mitochondrial membrane potential (ΔΨ_*m*_) constitutes an indicator of a cell’s capacity to generate ATP by oxidative phosphorylation, providing a reliable index of cell health or injury. Changes in this physiologic mitochondrial parameter can be monitored through confocal imaging. To do this, the cationic dye JC-1 was employed for cell staining. JC-1 accumulates in mitochondria in a potential-dependent manner, which results in an emission shift from green (~525 nm) to red (~590 nm) [29]. Therefore, JC-1 is an indicator of mitochondrial depolarization that allows the assessment of cell responses to an applied stimulus. In healthy cells with a normal (ΔΨ_*m*_), JC-1 enters and accumulates in the negatively charged mitochondria, forming red fluorescent J-aggregates.

In unhealthy cells, mitochondria are less negative because of increased membrane permeability and the consequent loss of electrochemical potential. As such, JC-1 does not reach a sufficient concentration to trigger the formation of J-aggregates, and it retains its original green fluorescence. Thus, evaluating the red/green fluorescence ratio of JC-1 in the mitochondria provides a direct assessment of the state of mitochondria polarization.

Cells stained with 1 µM JC-1 (Molecular Probes, Inc., Eugene, OR, USA) were incubated for 1 h in the dark (37 °C—5% CO_2_) before imaging. To quantify changes in the mitochondrial membrane potential, images were acquired with a Nikon A1-MP (NIKON CORPORATION Fuji Bldg., 2-3, Marunouchi 3-chome, Chiyoda-ku, Tokyo 100-8331, Japan) confocal microscope in two separated channels (excitation: 488 nm, emission 525/50 nm for the green channel, and excitation: 561 nm, emission: 595/50 nm for the red channel). The PMT gain and laser intensity were kept fixed for all the samples.

ΔΨ_*m*_ was calculated as Equation (1):

$${\Delta \psi }_{m}=\frac{I_{R}}{I_{G}}$$

where $I_{R}$ and $I_{G}$ are the fluorescence emission intensities in the red and green channel, respectively. The formation of more J-aggregates results in a red shift of the dye, reflecting a higher mitochondrial membrane potential. Whereas depolarization causes a decrease in the red/green fluorescence ratio, as a result of the formation of fewer J-aggregates. To avoid biases in the determination of the ratio, photomultipliers were kept at the same operative tension during the measurements.

### 2.6. Mitochondrial ROS Detection

Mitochondrial ROS production was determined using a Cayman Mitochondrial ROS Detection Assay Kit (catalogue number 701,600; Cayman Chemical, 1180 E. Ellsworth Road Ann Arbor, MI 48108, USA). ARPE-19 cells were seeded in a 96-well plate at a density of 1.5 × 10^4^ cells/well and treated according to the experimental design. After treatment, the culture media were carefully aspirated and 120 µL of pre-warmed cell-based assay buffer was added. Subsequently, the buffer was aspirated and 100 µL of mitochondrial ROS detection reagent staining solution was added to each well and the plate was incubated for an additional 20 min, protected from light, at 37 °C. At the end of the incubation period, the Staining Solution was removed and after washing with PBS, the samples were read with a CytoFluor multiwell plate reader (Victor3-Wallac-1420; PerkinElmer, Waltham, MA, USA), with an excitation wavelength of 480 nm and an emission wavelength of 560 nm. ROS production is expressed as fluorescence intensity and as a percentage relative to the control.

### 2.7. Mitochondrial Complex I–III Activity Measurements

Analysis of the activity of mitochondrial respiratory complexes (I-III) was performed on purified mitochondria [26]. Mitochondria were isolated using a mitochondria/cytosol fractionation kit (MBL, Medical & Biological Laboratories, 200 Dexter Ave., Watertown, MA 02472, USA). Pelleted cells (50 × 10^6^ cells), after incubation with 1 mL of cytosol extraction buffer mixture, were homogenized and centrifuged at 800× *g* for 1 min. The supernatant was collected and centrifuged at 15,000× *g* for 10 min at 4 °C. The resulting pellet was solubilized in a mitochondrial extraction buffer mixture. 

Respiratory Complex I (NADH: ubiquinone oxidoreductase), II (succinate dehydrogenase), and III (ubiquinol–cytochrome c oxidoreductase) activity was determined using specific assay kits (Cayman Chemical, Ann Arbor, MI, USA): MitoCheck^®^ Complex I Activity Assay Kit (#700930), MitoCheck^®^ Complex II Activity Assay Kit (#700940), and MitoCheck^®^ Complex III Activity Assay Kit (#700950). Complex I was determined by measuring the decrease in NADH oxidation, reflected as a decrease in absorbance at 340 nm, in the presence of antimycin A (10 M). Complex II activity was assessed by the rate of reduction of 2,6-dichlorophenol-indophenol (DCPIP), which is protonated by reduced coenzyme Q and is reflected by a decrease in absorbance at 600 nm. Mitochondrial complex III activity was determined by the rate of reduction of cytochrome c, which is reflected by an increase in absorbance at 550 nm. Respiratory complexes’ activities are expressed as percentage changes compared with the control (100%).

### 2.8. Measurement of Cytochrome C Release

ARPE-19 cells were plated in Petri dishes at a seeding density of 3 × 10^6^ cells/35 mm dish. At the end of the experiments, cells were harvested by centrifugation. The pellets were lysed in 50 μL of chilled cell lysis buffer and centrifuged at 10,000× *g* for 5 min at 4 °C. The cytochrome c presence was tested on supernatants using a sandwich ELISA assay (Mouse/Rat Cytochrome C SimpleStep ELISA^®^ Kit-Abcam, #ab210575; Abcam Biomedical Campus, Discovery Drive, Trumpington, Cambridge CB2 0AX, UK) according to the experimental protocol and the manufacturer’s instructions. The samples were read with a microplate reader at 450 nm (Victor3-Wallac-1420; PerkinElmer, Waltham, MA, USA). All values obtained are expressed as a percentage relative to the untreated control.

### 2.9. Measurement of Caspase-3 Activity

The supernatants obtained and as reported for cytochrome c, were also used to assess the caspase–3 assay. Caspase-3 activity was quantified using a specific colorimetric assay kit (Sigma Chemical Co.-St. Louis, MO, USA) according to the manufacturer’s protocol [30]. The protease activity was assessed using a plate reader (Victor3-Wallac-1420; PerkinElmer, Waltham, MA, USA) at a wavelength of 405 nm. The values obtained are expressed as a percentage relative to the untreated control.

### 2.10. Statistical Analysis

Each experiment was repeated at least two or three times. All experiments were performed with no fewer than eight replicates per experimental group. All the results are presented as the means ± SEM of (*n*) replicates per experimental group. Data were subsequently analyzed by one-way ANOVA with post hoc Newman–Keuls for comparisons between group means, or by Dunnett test, when appropriate, using a PrismTM computer program (GraphPad, San Diego, CA, USA). However, in the experiments carried out to evaluate the mitochondrial membrane potential, the *p*-value was determined by one-way ANOVA with post hoc Tukey’s test. 

Differences were considered statistically significant if *p* < 0.05.

## 3. Results

### 3.1. Idebenone Cytoprotective Effects

**Figure 1:**
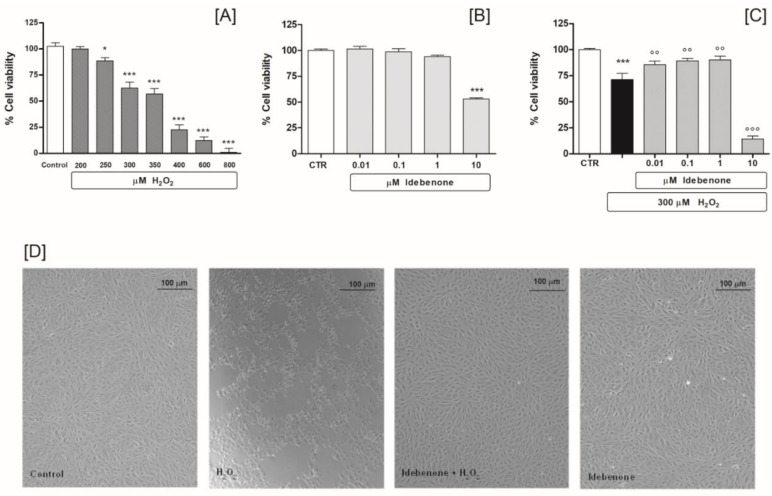
Idebenone pre-treatment diminishes H_2_O_2_–induced cell death. ARPE-19 cell viability: after treatment with different concentrations of H_2_O_2_ (range 200–800 µM) for 24 h (**A**); after idebenone (range 0.01–10 µM) pre-treatment for 24 h, both under baseline conditions (**B**); and following stimulation with 300 µM H_2_O_2_ (**C**). Phase-contrast micrography shows morphological changes of ARPE-19 cells treated for 24 h with 300 µM H_2_O_2_ in the absence or presence of idebenone (1 µM) pre-treatment (24 h), compared to the control. Image of idebenone alone is also shown, (**D**). The results are from two independent experiments, each including five replicates per experimental group. Data are expressed as a percentage relative to the untreated cells (control = 100%), the means ± SEM of 10 replicates per group. * *p* < 0.05 and *** *p* < 0.001 vs. control; °° *p* < 0.01 and °°° *p* < 0.001 vs. H_2_O_2_ alone.

In designing our experimental protocol, we initially planned to use dose-response experiments with H_2_O_2_ (range 200–800 μM) in order to determine the cell growth inhibition rate in ARPE-19 cells, using an MTS assay. In our design tests, the results showed a directly proportional relationship between the concentration of H_2_O_2_ and cell growth inhibition. In particular, the concentration of 300 µM inhibited growth by about 60% (62.6 ± 14.9 *n* = 8), after 24 h of treatment. Therefore, this dose was included in our experimental protocol to induce cytotoxicity and simulate a oxidative damage (Figure 1A). Next, we conducted an experimental design review of dose-response studies, to determine IDB’s cytoprotective effects under basal and oxidative stress conditions. We found that a 24 h treatment of IDB (range 0.01–1 μM) does not modify ARPE-19 cell viability under basal conditions. However, the highest dose administered (10 μM) has proven to be toxic, reducing cell viability by approximately 47% (Figure 1B). Exposure of ARPE-19 cells to 300 μM H_2_O_2_ for 24 h reduced cell viability, which was counteracted by pre-treatment (24 h) with IDB. In fact, the substance was able to antagonize the cellular oxidative stress induced by H_2_O_2_ in a dose-dependent manner (Figure 1C). 

The results were also supported by the morphological observations performed at the end of the experiments (Figure 1D). Interestingly, the higher dose of IDB (10 μM) resulted in remarkable oxidative damage compared to cells exposed to the H_2_O_2_ alone (Figure 1C). Consequently, 300 µM H_2_O_2_ and 1 µM IDB were set as the study protocol concentrations.

### 3.2. Idebenone Stimulates Nuclear Activation of NRF2 and Modulates the Bcl-2/Bax Ratio

**Figure 2:**
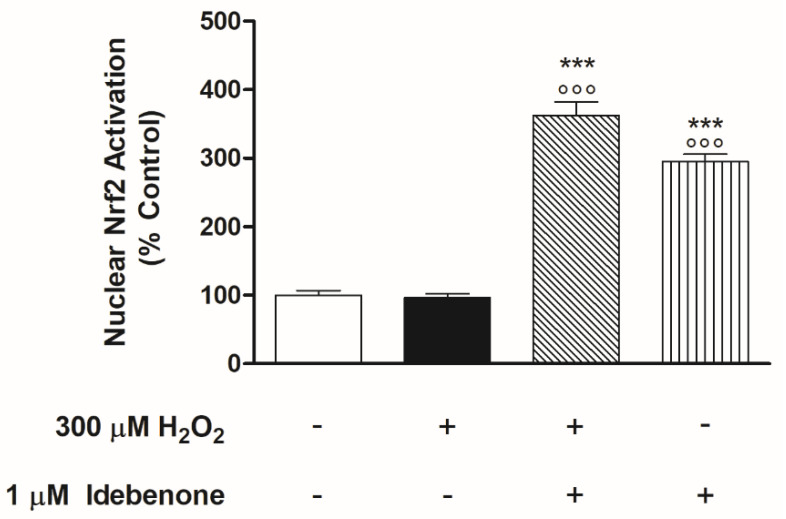
Idebenone stimulates Nrf2 nuclear translocation in ARPE-19 cells. Idebenone significantly increased NRF2 nuclear activation in both basal and 300 μM H_2_O_2_ stimulated conditions. All of the optical density values (see Materials and Methods) are expressed as a percentage relative to the non-treated cells (control 100%) ± SEM of three independent experiments performed in triplicate (nine in all, for each experimental group). *** *p* < 0.001 vs. control; °°° *p* < 0.001 vs. H_2_O_2_ alone.

We were interested in investigating whether IDB’s cytoprotective effects somehow correlated with Nrf2 (nuclear factor-erythroid factor 2-related factor 2) signaling pathway activation. Nrf2 is a transcription factor controlled by redox-dependent modifications and is essential for the regulation of genes responsive to different types of stress, including oxidative stress [31]. This regulatory mechanism is based on the nuclear availability of Nrf2 itself [32]. Therefore, we investigated whether IDB was able to modulate the nuclear translocation of Nrf2. To elucidate this, we assessed Nrf2 activation in the nuclear fraction of the cells after H_2_O_2_ treatment with or without 1 µM IDB pre-treatment with IDB. The pre-treatment with IDB showed remarkable nuclear translocation of Nrf2 both in baseline and stimulated conditions (Figure 2). Nuclear levels of Nrf2 in cells exposed to H_2_O_2_ alone were not different from those of the untreated controls (Figure 2). 

**Figure 3:**
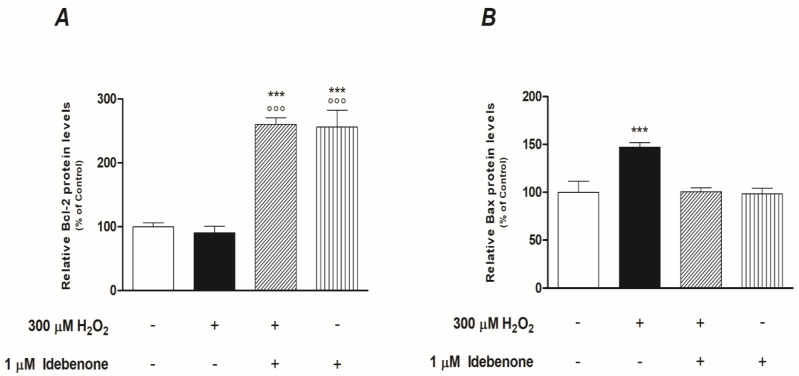
Idebenone effect on the protein levels of Bcl-2 and Bax. Idebenone pre-treatment promotes Bcl-2 expression (**A**) but inhibits Bax expression (**B**) after H_2_O_2_ induced oxidative stress for 24 h. Both histograms represent the means ± SEM of two independent experiments performed in quadruplicate (eight replicates per experimental group). *** *p* < 0.001 vs. control; °°° *p* < 0.001 vs. H_2_O_2_ alone.

The switching on and off of Nrf2 protects cells from oxidative/apoptotic damage induced by endogenous and exogenous harmful factors by transcriptional activation of genes that control cellular homeostasis. Our study focused on NRF2-mediated IDB anti-apoptotic effects, particularly those under direct mitochondrial control. Consequently, we conducted experiments to verify whether IDB modulated the intracellular levels of two antagonist proteins of the Bcl-2 family, Bax and Bcl-2. We observed high intracellular levels of Bax and low levels of Bcl-2 (the values are superimposable with those of the untreated control) after exposition to oxidative stress alone (Figure 3). Pre-treatment (24 h) with 1 µM IDB significantly increased both basal and stimulated Bcl-2 levels, while antagonizing those of Bax, in the presence of H_2_O_2_; IDB did not change baseline Bax levels (Figure 3).

### 3.3. Idebenone Supports Mitochondrial Functions in Presence of Oxidative Stress

Being able to modulate the permeability of the outer mitochondrial membrane, the proteins of the Bcl-2 family play a central role in the activation of the “intrinsic mitochondrial” pathway of apoptosis. Therefore, we sought to understand if the pretreatment with IDB could be supportive to mitochondria under conditions of oxidative stress. To this end, the m, mitochondrial ROS levels, and respiratory chain complexes activation were examined. 

**Figure 4:**
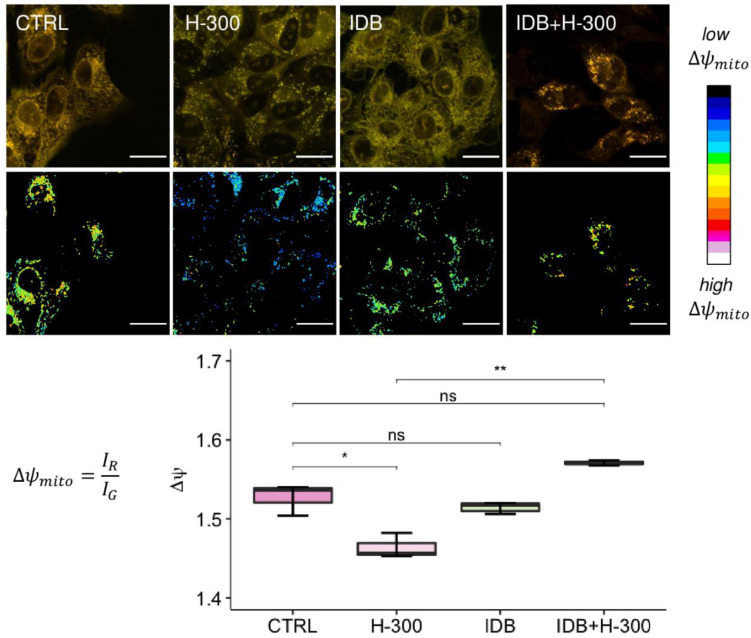
The role of idebenone against H_2_O_2_-induced mitochondrial depolarization. The panel shows, in the first row, representative images of ARPE-19 cells stained with JC-1 under different conditions (untreated, CTRL; 300 µM H_2_O_2_, H-300; Idebenone, IDB; and pre-treated with idebenone 24 h before H_2_O_2_, IDB + H-300), together with the ratio maps of mitochondrial membrane potential, in the second row. In the composite confocal images, the fluorescence signal from monomers is represented in green (emission: 525/50 nm), while the red channel (emission: 595/50 nm) collects the signal from aggregates. In the mitochondrial membrane potential maps, each pixel is colored according to the red/green fluorescence intensity ratio, with black pixels representing low R/G values (i.e., mitochondrial depolarization) and white pixels representing high values of the R/G ratio (i.e., mitochondrial hyperpolarization). The scale bar is 20 µm. The distribution of the R/G values, corresponding to the mitochondrial membrane potential, are reported in the box plot (y-axis) for each sample (x-axis). One-way ANOVA analysis was carried out with post hoc Tukey’s test for multiple group comparisons (* *p* < 0.05; ** *p* < 0.01; ns = not statistically significant).

Initially, we investigated if IDB was able to antagonize the dissipation of mitochondrial membrane potential induced by oxidative stress and which is linked to the cytoplasmic release of pro-apoptotic proteins. Mitochondrial membrane potential (Δψm) changes were determined through confocal microscopy imaging, after cell staining with the ratiometric cationic dye JC-1 (see Materials and Methods). Composite dual-channel images, together with maps of the red/green fluorescence intensity ratio, namely, the m, are represented in the panel in Figure 4, for untreated cells (CTRL, first column), cells treated with 300 µM hydrogen peroxide (H-300, second column), cells treated with IDB (IDB, third column), and cells treated with IDB 24 h before H_2_O_2_ (IDB + H-300, fourth column). Treating cells with 300 µM H_2_O_2_ induced a strong mitochondrial depolarization, which resulted in a shift towards the blue in the representative ratio maps. The addition of IDB in the presence of high H_2_O_2_ levels increased the mitochondrial membrane potential, restoring the physiological conditions.

The box plot in Figure 4, displays the distribution of mitochondrial membrane potential for untreated ARPE-19 cells (CTRL, in dark pink), cells treated with 300 µM H_2_O_2_ (H-300, in light pink), cells pre-treated (24 h) with IDB and afterward incubated for further 24 h with 300 µM H_2_O_2_ (IDB + H-300, in dark green), as well as for cells treated with 1µM IDB alone (IDB, in light green). When comparing the distribution of the mitochondrial membrane potential Δψm between the untreated (CTRL) and the H_2_O_2_ treated (H-300) cells, a significant decrease was observed (*p*-value = 0.014). However, pre-treatment with IDB in the presence of H_2_O_2_ preserved mitochondrial functionality, resulting in a significant increase in membrane polarization compared to H_2_O_2_ alone (*p*-value = 0.0036) and with approximately superimposable values of Δψm compared to the untreated cells (*p*-value = 0.055). The administration of IDB alone had no effect on mitochondrial membrane potential (*p*-value = 0.39 vs. CTRL).

**Figure 5:**
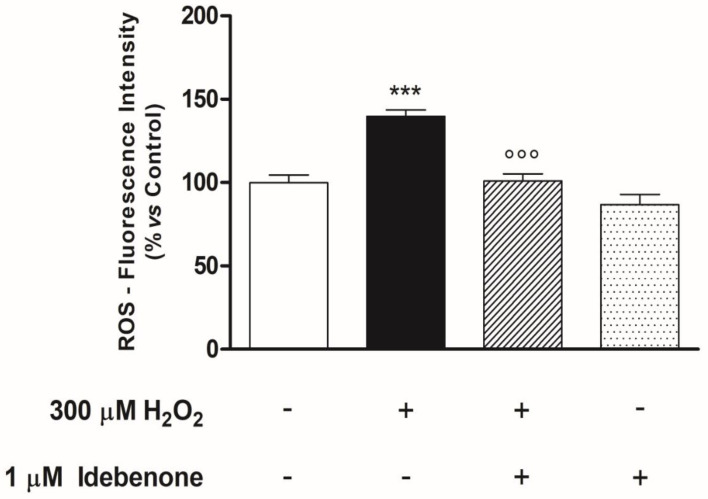
Idebenone reduces mitochondrial ROS production in ARPE-19 cells treated with H_2_O_2_. Idebenone pre-treatment (24 h) blocks H_2_O_2_-induced mitochondrial ROS accumulation. Mitochondrial ROS levels are quantified by measurement of DCF fluorescence (see Materials and Methods) and the results are expressed as a percentage of the untreated control (control = 100%). Results are from two independent experiments, the means ± SEM of 10 replicates per group. *** *p* < 0.001 vs. control; °°° *p* < 0.001 vs. H_2_O_2_ alone.

Oxidative damage-induced mitochondrial dysfunction is characterized not only by the dissipation of ΔΨ_*m*_ but also by the excessive production of ROS and the alteration of the mitochondrial respiratory chain complexes. Therefore, the subsequent experiments in our study were designed to investigate the effect of the IDB on these parameters. In the experimental conditions, H_2_O_2_-induced oxidative stress resulted in a significant increase in mitochondrial ROS levels, which was antagonized by pre-treatment with IDB for 24 h. However, IDB did not change mitochondrial ROS concentrations in the absence of an oxidative stimulus (Figure 5).

**Figure 6:**
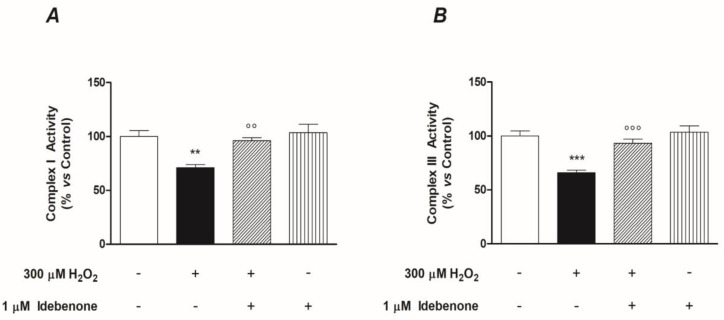
Idebenone supports the functions of mitochondrial respiratory chain complexes (I and III). The graphs show the ability of the idebenone pre-treatment (24 h) to restore complex I (**A**) and complex III (**B**) mitochondrial respiratory functions, which were depressed after oxidative stress induced by 24 h of treatment with H_2_O_2_. Both graphs are the result of two independent experiments performed in quintuplicate (*n* = 10/group). The results are expressed as a percentage of the untreated control (control = 100%). Results are from two independent experiments, the means ± SEM of 10 replicates per group. ** *p* < 0.01 and *** *p* < 0.001 vs. control; °° *p* < 0.01 and °°° *p* < 0.001 vs. H_2_O_2_ alone.

In addition, our results showed a support mechanism for the mitochondrial respiratory chain, linked to IDB’s effects as previously observed. IDB pre-treatment significantly antagonized the H_2_O_2_-induced depressing effects on the activity of complexes I and III of the respiratory chain, whereas it had no impact under basal conditions (Figure 6). We did not observe any modification related to complex II (data not shown).

### 3.4. Idebenone Inhibits Cytochrome C-Mediated Caspase-3 Activation

**Figure 7:**
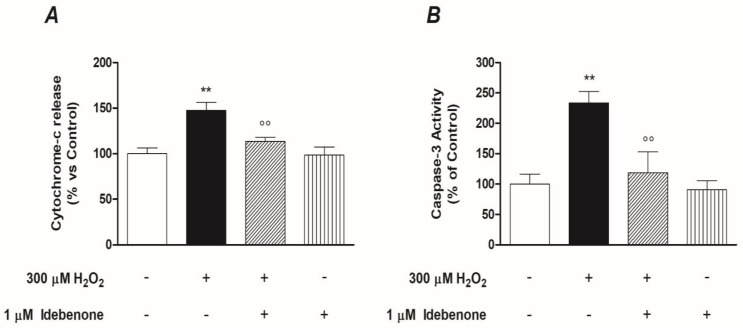
Idebenone inhibits cytochrome c release and Caspase-3 enzymatic activity. Effect of the idebenone pre-treatment (1 µM for 24 h) both on the release of Cytochrome-c (**A**) and the activity of Caspase-3 (**B**) in the presence or absence of 300 µM H_2_O_2_-induced oxidative stress for a further 24 h. Results are quantified by measurement of OD (see Materials and Methods) and are reported as a percentage of the ARPE-19 untreated cells (arbitrarily set to 100%). The graphs are the result of three independent experiments performed in triplicate, the means ± SEM of 9 replicates per group. ** *p* < 0.01 and °° *p* < 0.01 vs. control and H_2_O_2_ alone, respectively.

At this point of the study, to confirm the results previously observed, we investigated the role of IDB in the regulation of the cytochrome-c release and the Caspase-3 activation, downstream events of the intrinsic apoptotic pathway. Since the release of mitochondrial cytochrome-c is believed to be one of the first events linked to the dissipation of mitochondrial potential (ΔΨ_*m*_), we initially investigated whether the pre-treatment with IDB modulated the output of cytochrome-c from mitochondria with or without oxidative stress. For this purpose, the ARPE-19 cells were pre-treated with IDB for 24 h and were subsequently treated with 300 µM H_2_O_2_ for a further 24 h. Under these conditions, the treatment with 1 µM IDB inhibited significantly the release of cytochrome-c in the cytoplasm (Figure 7A). These results were consistent with those obtained in the subsequent experiments, in which we studied the activity of Caspase-3 after oxidative stress, in the presence or absence of pre-treatment IDB. As shown in Figure 7B, 1 µM IDB antagonized in a significant manner the Caspase-3 activity in ARPE-19 cells exposed to 300 µM H_2_O_2_. However, IDB did not modify either the release of Cytochrome-c or the activity of Caspase-3 in basal conditions (Figure 7A,B).

## 4. Discussion

Idebenone (IDB) is a synthetic variant of coenzyme Q10 (CoQ10), an essential cofactor of the mitochondrial electron transport chain. The structures of the two substances are partially related, although IDB has a shorter “tail” attached to the quinone fraction, which gives it better pharmaco-chemical characteristics compared to its archetype. These properties allow it to exercise its peculiar function of high-energy electron shuttle at the level of the mitochondrial respiratory chain [2,33]. It is assumed that by interacting with this transport mechanism, IDB increases the production of ATP necessary for mitochondrial functioning, reduces free radicals, inhibits lipid peroxidation, and, consequently, protects the lipid membrane and mitochondria from oxidative damage [1,34]. Therefore, since its introduction in the pharmaceutical market in the 1980s, IDB has been classified as a psychostimulant and nootropic drug. [35]. In effect, pre-clinical and clinical studies have shown that IDB improves learning times and memory activity (this positive effect is linked to the synthesis and turnover of serotonin and acetylcholine) [36,37], as well as its efficacy in mental deterioration syndromes of various origins, characterized by cognitive-behavioral disorders (e.g., Alzheimer) [38,39]. In more recent times, IDB has also been used to treat several rare hereditary diseases characterized by mitochondrial damage, such as Duchenne muscular dystrophy and Friedreich’s ataxia [8,40,41,42]. However, in some cases, desired outcomes were lower than expected. Today, IDB represents an important and successful alternative in the treatment of Leber’s hereditary optic neuropathy (a rare mitochondrial disease), and its approval as a drug has opened new and interesting scenarios for its use in eye diseases characterized by mitochondrial alterations. [43,44,45]. The in vitro findings presented in this paper are consistent with this hypothesis. The evidence that has emerged from our studies suggests that IDB shows cytoprotective effects on RPE cells, modulating apoptosis and supporting mitochondrial functionality in the presence of oxidative damage. 

The retinal pigment epithelium (RPE) is constituted by a monolayer of pigmented cells on the outside of the sensorineural retina. These cells are a type of energy-intensive cell, and the mitochondria are the “power plant” and the main endogenous source of ROS within them. However, RPE is particularly vulnerable to the toxicity of high ROS concentrations present during oxidative stress, as well as to the mitochondrial dysfunction that appears to be an early event in the process of cell death induced by H_2_O_2_ [18]. It has been shown previously that IDB has a cytoprotective effect on RPE cells after short-term oxidative stress exposure; an effect characterized by a reduction of intra-cytoplasmic ROS levels and the normalization of the Bax/Bcl-2 ratio, which are both altered in cells exposed to H_2_O_2_ [23]. Starting from this assumption, and using a similar but non-overlapping experimental model, consisting of a pre-treatment for 24 h with IDB and subsequent exposure of the cells (a human cell line; ARPE-19) to H_2_O_2_ for a further 24 h, we have shown that IDB can have pleiotropic effects on RPE cells exposed to H_2_O_2_ oxidative stress and which have H_2_O_2_-induced oxidative damage. We hypothesize that the cytoprotective effect of IDB involves the transcription factor Nrf-2, which induces the transcription of genes that encode both antioxidant proteins and phase 2 enzymes implicated in the detoxification of xenobiotics. Therefore, Nrf-2 is a fundamental transcription factor in maintaining cellular redox homeostasis. In basal conditions, Nrf-2 is kept in the cytoplasm by Keap-1, which represents its main negative regulator. However, in the presence of electrophilic or oxidative stress, Nrf-2 detaches from Keap-1, and once free, it can translocate inside the nucleus, where, through the formation of a heterodimer, it binds to the ARE sequences present on DNA, activating the transcription of its target antioxidant genes [46,47]. Our findings suggest that pre-treatment with IDB stimulates the Nrf-2 nuclear translocation and the subsequent transcription of its target genes, in response to H_2_O_2_-induced oxidative stress. International scientific literature evidence in other experimental models supports these data. For instance, in patients with Friedreich’s ataxia, IDB induces the expression of Nrf-2 and then stimulates the transcription of its target genes in the fibroblasts. Interestingly, IDB showed a lower degree of activation of the antioxidant genes induced by Nrf-2 than the drugs to which it was compared [48]. In addition to the Nrf-2 nuclear translocation modulating the expression of various antioxidant, detoxifying and drug transporter proteins can also regulate the expression and induction of genes of the Bcl-2 family. In particular, the nuclear overexpression of Nrf-2, mediated by antioxidants, up-regulates the antiapoptotic protein Bcl-2 and down-regulates the proapoptotic protein Bax [49]. Following this reasoning, we characterized the cytoprotective role of IDB on ARPE-19 cells exposed to H_2_O_2_, confirming the ability of IDB to increase the levels of Bcl-2 and decrease those of Bax, whilst at the same time highlighting its role as a regulator of mitochondrial functions by activating the intrinsic mitochondrial pathway of apoptosis. The Bcl-2 family proteins control the intrinsic mitochondrial pathway of apoptosis by regulating the mitochondrial outer membrane permeabilization through a complex network of protein–protein and protein–membrane interactions. In the presence of oxidative damage, the balance between cell survival and death is broken, resulting in an increase of pro-apoptotic (e.g., Bax) protein activity compared to anti-apoptotic (e.g., Bcl-2) protein activity, with a consequential increase in mitochondrial membrane permeability and the release of cytochrome c. [50,51].

In our study, pre-treatment with IDB maintained mitochondrial membrane potential at physiological values, antagonizing membrane potential dissipation induced by H_2_O_2_, in addition to increasing BCL-2 levels, thereby confirming the initial hypothesis of a modulation of the intrinsic pathway of apoptosis by IDB. The reduction in mitochondrial ROS levels and the maintenance of the activity of the mitochondrial respiratory chain complexes, observed after pre-treatment with IDB in response to oxidative damage induced by high concentrations of H_2_O_2_, reinforced this hypothesis. However, some studies, conducted on isolated mitochondrial complex or on mitochondrial fractions, have shown that IDB, at high doses, inhibits complex I and promotes superoxide production [1,2,52]. Of course, it cannot be excluded a priori that the effects at the mitochondrial level observed in our study are linked to other IDB mechanisms, highlighted in other experimental models, such as: (a) NQO1-dependent cytosolic-mitochondrial electron transfer capacity [34,53]; (b) inhibition of lipid peroxidation and stabilization of the mitochondrial membrane [54]; and (c) the increase in the activity of antioxidant genes such as superoxide dismutase (SOD), catalase (CAT), and glutathione peroxidase (GSH-Px) [55]. However, our results, which demonstrate that pre-treatment with IDB, in presence of H_2_O_2_-induced oxidative damage, can antagonize both the release of cytochrome c from mitochondria and the activation of caspase-3 (both in the downstream, critical steps, of intrinsic apoptosis), provide verification that our hypothesis is realistic.

## 5. Conclusions

In conclusion, this study confirms and expands on previous results that report that Idebenone (IDB) is a substance capable of protecting The retinal pigment epithelium (RPE) cells from oxidative damage. In particular, we hypothesize that IDB carries out its cytoprotective action by modulating the intrinsic pathway of apoptosis, after directly stimulating the nuclear translocation of NRF2 and the subsequent synthesis of Bcl-2. In light of these observations, IDB should be included in the large group of antioxidant/antiapoptotic substances that appear to be promising tools in the management of ocular epitheliopathies and related disorders. Among these, it seems to have promising applications in maculopathies where RPE, through exposure to external environmental factors and normal metabolic processes, is subjected to continuous oxidative stress which, in the long run, causes oxidative damage, characterized by an excessive formation of ROS in the cellular organelles, mostly at the mitochondrial level. Although the experimental design adopted here does not allow for definitive conclusions to be drawn, it can assist in the planning of new preclinic studies aimed at a better understanding of IDB’s therapeutic efficacy and its mechanism of action in the treatment of various retinal degenerative diseases, characterized by age-related mitochondrial dysfunction.

## Data Availability

Data supporting reported results are available on request from the corresponding author.
